# MMP1 Overexpression Promotes Cancer Progression and Associates with Poor Outcome in Head and Neck Carcinoma

**DOI:** 10.1155/2022/3058342

**Published:** 2022-09-05

**Authors:** Wei Zhang, Xinghong Huang, Rong Huang, Hong Zhu, Pu Ye, Xuyang Lin, Shuangyue Zhang, Meng Wu, Feng Jiang

**Affiliations:** ^1^The Affiliated Huaian No.1 People's Hospital of Nanjing Medical University, Huaian, 223300 Jiangsu Province, China; ^2^School of Medical Technology, Taizhou Polytechnic College, Taizhou, 225300 Jiangsu Province, China

## Abstract

Matrix metalloproteinase-1 (MMP1) has been reported to play key roles in a variety of cancers by degrading the extracellular matrix. However, its carcinogenic roles have not been shown yet in head and neck squamous cell carcinoma (HNSCC). This study aimed to elucidate its expression pattern and functional roles as well as clinical significance in HNSCC. The Cancer Genome Atlas (TCGA), Gene Expression Omnibus (GEO), and immunohistochemistry (IHC) were utilized to determine the MMP1 expression pattern and the associations between its expression and patients' outcome in HNSCC. Mice tongue squamous cell carcinoma model induced by 4-nitroquinoline 1-oxide (4NQO) and siRNA-mediated cellular assay in vitro were utilized to evaluate the oncogenic role of MMP1. The biological functions and cancer-related pathways involved in MMP1-related genes were found through bioinformatics analysis. Both mRNA and protein abundance of MMP1 were highly increased in HNSCC as compared to its non-tumor counterparts. MMP1 overexpression positively correlated with advanced tumor size, cervical node metastasis, and advanced pathological grade and lower patients' survival. In the 4NQO-induced animal model, MMP1 expression increased along with the progression of the disease. In HNSCC cells, siRNA-mediated knockdown of MMP1 significantly inhibited cell proliferation, migration, and invasion and activated apoptosis and epithelia-mesenchymal transition (EMT). GSEA, GO, and KEGG analyses showed that MMP1 expression was significantly related to cancer-related pathways and cancer-related functions. Together, our results demonstrated MMP1 serves as a novel prognostic biomarker and putative oncogene in HNSCC.

## 1. Introduction

Head and neck squamous cell carcinoma (HNSCC) is the ninth common cancer worldwide with more than 550000 new cases and 300000 death cases [1]. Risk factors for HNSCC include smoking, drinking, chewing areca nuts, and infection with human papillomavirus. Treatment options for HNSCC include surgical resection, chemotherapy, and focused radiation therapy. Nevertheless, the 5-year survival rate of HNSCC patients has not been significantly improved due to cervical lymph node metastasis, locoregional recurrence, and therapeutic resistance [2, 3]. In addition, traditional predictive parameters such as clinical stage, depth of invasion, surgical margin, and involvement of cervical lymph nodes increasingly unable to meet the clinical needs [4]. Despite extensive study into HNSCC, the underlying cellular and molecular mechanism of HNSCC emerge remains incompletely understood. Therefore, the exploration of molecular mechanisms underlying HNSCC will aid in the development of molecular targeted therapies for this dreaded disease.

Molecular biology research and whole genome sequencing technology provide rich resources for developing biomarkers that will be utilized for early diagnosis, patient stratification, personalized treatment, and prognosis prediction [5, 6]. Matrix metalloproteinases (MMPs) are a family of endopeptidases, which are composed of more than 28 kinds of human MMPs targeting the degradation and proteolytic process of components of the extracellular matrix. In addition to being vital for development, wound healing, and inflammatory diseases, MMPs are also important in the initiation and progression of tumors [7, 8]. By degrading the extracellular matrix (ECM), they regulate the behavior of various types of cells relevant to cancer biology [9]. Accumulating evidence has shown MMPs play essential roles including tumor growth, differentiation, apoptosis, angiogenesis, migration, invasion, and immune surveillance in multiple stages of cancer [8, 10]. As a crucial member of the MMP family, the aberrant expression of matrix metalloproteinase-1 (MMP1) has involved in various behaviors of cancer. Elevated expression of MMP1 and its prognostic value have been revealed in colorectal cancer [11], cervical squamous cell carcinoma [12], uveal melanoma [13], and colon cancer [14]. In addition, MMP1 has been identified as an oncogene facilitating the proliferation and migration of cutaneous squamous cell carcinoma (CSCC) via the activation of Wnt pathway [15]. Complementally, downregulation of MMP1 inhibits the progression of colorectal cancer by suppressing the PI3K/Akt/c-myc signaling pathway and epithelial-mesenchymal transformation (EMT) [11]. In esophageal squamous cell carcinoma (ESCC) and HNSCC, decreased MMP1 inhibited tumor invasion and metastasis in vitro and vivo [16, 17]. Epithelial-mesenchymal transformation (EMT) has been considered a necessary regulatory mechanism for the migration and invasion of malignant tumors in recent years [18]. The latest investigations indicate that MMP1 plays a vital role in EMT in colorectal cancer and squamous cell carcinoma of the skin [11, 19]. Nevertheless, the precise biological functions of MMP1 in HNSCC are just now beginning to surface in selected contexts and the associations between MMP1 and EMT remain yet unexplored in HNSCC.

The above results suggest that MMP1 may be a new potential oncogene in human cancer. Nevertheless, the role of MMP1 in the development of HNSCC remains unclear. In the present study, we investigated the expression of MMP1 in HNSCC and revealed the correlations between its expression and clinical parameters by collecting data from TCGA, GEO, and our samples. Additionally, small interference RNA and 4NQO-induced animal model were utilized for exploring the tumorigenic roles of MMP1 in HNSCC. Finally, much genomic, epigenomic, and transcriptomic data are available in online databases in the era of big data. This study used bioinformatic methods to detect the biological functions of MMP1-related genes from public databases. All these findings indicate MMP1 as a putative oncogene involving in HNSCC initiation and progression and as a novel prognostic biomarker utilizing for HNSCC.

## 2. Materials and Methods

### 2.1. Patients' Specimens and Immunohistochemical Staining (IHC)

A total of 103 patients diagnosed with HNSCC from Jan. 2010 to Dec. 2017 underwent radical resections at the Department of Oral and Maxillofacial Surgery, Affiliated Huaian No.1 People's Hospital of Nanjing Medical University. Patient inclusion criteria were described as follows: (1) primary HNSCC with no prior chemotherapy or radiotherapy; (2) patients underwent radical tumor resection and neck lymph node dissection; (3) detailed demographic, clinical, pathological, and follow-up data available. The archived tissue samples and hematoxylin–eosin stained sections of each patient were retrieved. The previous histological diagnosis as SCC was further conformed according to the established histological criteria. The normal oral mucosa of 20 patients with non-cancer surgery was also enrolled as the control group. This study protocol was reviewed and approved by the Research Ethics Committee of the Affiliated Huaian No.1 People's Hospital. All included patients have signed written informed consent.

### 2.2. Immunohistochemical Staining and Scoring

Immunohistochemical staining for MMP1 was performed on 4 *μ*m-thick slides from formalin-fixed paraffin-embedded samples using routine procedures. Negative controls without primary MMP1 antibody (1 : 200; ab137332; Abcam) incubation were included. Immunoreactivity was semi-quantitatively evaluated according to staining intensity and distribution using the immunoreactive score which was calculated as intensity score × proportion score as we reported previously [20]. Intensity score was defined as 0, negative; 1, weak; 2, moderate; and 3, strong, while the proportion score was defined as 0, negative; 1, <10%; 2, 11–50%; 3, 51–80%; and 4, >80% positive cells. The total score ranged from 0 to 12. Accordingly, the immunoreactivity of each slide was categorized into three subgroups based on the final score: 0, negative; 1–4, low expression; and 4–12, high expression as we reported before [20].

### 2.3. Cell Culture and Small Interference RNA (siRNA) Transfection

Human Oral Keratinocytes (HOK), Fadu, Cal27, HN6, and HN4 were purchased from American Type Culture Collection (ATCC, Manassas, VA, USA). Cultures were carried out at 37°C and 5% CO2 in DMEN/F12 (Invitrogen) supplemented with 10% heat-inactivated fetal bovine serum (Gibco). The siRNA including MMP1 siRNA-1 (5′-ACACAAGAGCAAGATGTGG-3′), MMP1 siRNA-2 (5′-TAAGTACTGGGCTGTTCAG-3′), and control siRNA were purchased from GenePharma (Shanghai, China). Cells were transfected with MMP1 siRNA with final concentration 100 nM according to Lipofectamine 3000 instruction (Invitrogen, USA). Then, these cells were utilized for further experiments for 48 h.

### 2.4. RNA Extraction and Real-Time RT-PCR

According to the instructions of Trizol reagent (Invitrogen), using Trizol to dissolve cells and tissues and placing them on the ice box. Then adding an appropriate amount of chloroform to the RNA extract and centrifuge. The aqueous phase layer was obtained, and isopropanol was added again for centrifugation. Sucking the upper solution, then adding ethanol to dissolve the precipitate and centrifuge, and finally adding RNase free water to dissolve the RNA precipitate. Then, total RNA was reversely transcribed into first strand cDNA using PrimeScriptTM RT reagent kit (Takara). The generated cDNA was used for real-time PCR reaction using SYBR Premix Ex TaqTM kit (Takara) following the supplier's instructions. The primers were listed as follows: MMP1 (forward: AAATGCAGGAATTCTTTGGG, reverse: ATGGTCCACATCTGCTCTTG), N-cadherin (forward: TTTGATGGAGGTCTCCTAA, reverse: ACGTTTAACACGTTGGAA), E-cadherin (forward: CGAGAGCTACACGTTCACGG, reverse: GGGTGTCGAGGGAAAAATAGG), Vimentin (forward: AAAACACCCTGCAATCTTTCAGA, reverse: CACTTTGCGTTCAAGGTCAA), Snail (forward: ACACGGACAGGATTGACAGA, reverse: GCATGAAGAAGCCGCGAAGTGT), and GAPDH (forward: AGGTGAAGGTCGGAGTCAAC, reverse: AGTTGAGGTCAATGAAGGGG).

### 2.5. Western Blot Analysis

The proteins of each group were collected, and the precipitate was separated by lysis, shock, and centrifugation. The precipitate was separated by electrophoresis in 10% SDS-PAGE gel then transferred to PVDF membrane. Following 5% non-fat milk or BSA blocking, the membranes were incubated with related primary antibodies at 4°C overnight. Then, the membranes were incubated with secondary antibodies for 1 h and imaged by ECL chemiluminescence kit (Bio-Rad). The antibodies utilized as follows: MMP1 (1 : 1000; ab137332; Abcam), Vimentin (1 : 2000, #5741, CST), Snail (1 : 1000, #3879, CST), E-cadherin (1 : 2000, #3195, CST), N-cadherin (1 : 1000, #13116, CST), and GAPDH (1 : 2000 dilution, #2118, CST).

### 2.6. CCK-8 and Colony Formation Assay

For CCK-8 assays, the proliferation of HNSCC cells was assessed by absorbance using a CCK-8 cell viability assay (Cell Counting Kit8, Dojindo, Japan) according to the manufacturer's instructions. Cells were seeded in 96-well microplates at a density of 2 × 10^3^ cells per well. Cells were incubated in new medium containing 10% CCK-8 reaction solution. After incubation for 2 h, the absorbance was measured on a spectrophotometer microplate reader (Multiskan MK3, Termo) at a wavelength of 450 nm. For colony formation assays, the HNSCC cells were seeded in 6-well plates at a density of 1 × 10^3^ cells per well. The culture was terminated when the cells formed obvious clones under the microscope and then cells were fixed with paraformaldehyde for 30 minutes. The images were taken by the scanner.

### 2.7. Flow Cytometric Assay

The cells of each group were collected and made into single cell suspension. The cells were stained according to the instructions of Annexin V: PI Apoptosis Detection Kit (BD Bioscience). Apoptosis percentages were then detected using a FACSC aliber fow cytometer (BD Biosciences) and analyzed by Flowjo V10.1.

### 2.8. Wound Healing Assay and Invasion Assay

Cal27 cells were seeded in 2×105 cell/well in six-well plates. Then, we utilized pipette tip to create an artificial wound on the confluent cell monolayer. The suspended cells were washed with PBS for three times. Then, cells were cultured in medium with 1% FBS (Gibco). The wounds were photographed at 0, 24 h as indicated. For cell invasion assays, 5 × 10^4^ cells were placed into the upper chamber precoated with Matrigel and culture medium with 8% FBS was added to the lower chamber. After incubation for 24 h, non-migrated cells were removed with a cotton swab. The chambers were fixed with 4% paraformaldehyde for 15 min at room temperature and incubated with 0.1% crystal violet solution for 30 min at 37°C. Cells were determined under a light microscope.

### 2.9. Chemical-Induced HNSCC Animal Model

A well-known chemical-induced mouse model was utilized to further explore the expression of MMP1 in the occurrence and development of HNSCC. 6-week-old C57BL/6 mice were fed with water containing 50 *μ*g/mL 4NQO for 16 weeks and then fed with normal water for another 8-10 weeks. During these 8-10 weeks, the lesions of mouse tongue were observed every week. Mice that had been drinking normal water were used as control. The tongue specimens of mice at 16, 20, and 24 weeks were collected for histopathological analysis. This study protocol was reviewed and approved by the Research Ethics Committee of the Affiliated Huaian No.1 People's Hospital.

### 2.10. Bioinformatics Analysis of MMP1

MMP1 mRNA expression level and correlated clinicopathological data of HNSCC were obtained from 2 public datasets: TCGA (The Cancer Genome Atlas) (https://cancergenome.nih.gov/) and GEO (Gene Expression Omnibus) (https://www.ncbi.nlm.nih.gov/gds/). MMP1 mRNA expression levels (log2-transformed) in HNSCC and normal counterparts were retrieved and statistically compared. The associations between expression status of MMP1 mRNA (high or low using median value as cutoff) and patient survival were determined by the Kaplan-Meier analysis which was downloaded from GEPIA2 (http://gepia2.cancer-pku.cn/). MMP1-correlated gene chart (.tsv) was obtained from cBioPortal (https://www.cbioportal.org/), and genes with Spearman's correlation >0.2 was filtered for next step analysis. Median cutoff was used to define the patients from TCGA-HNSCC cohort who has the high/low MMP1 expression then differentially expressed genes (DEGs) between these two groups which further served as the source for Gene Set Enrichment Analysis (GSEA), Gene Ontology (GO), and KEGG pathway. This analysis was performed on R packages ClusterProfiler [21].

### 2.11. Statistical Analysis

All data were performed using GraphPad Prism 8, R packages ggplot2, or SPSS 21.0 software. All the experiments were repeated three times, then Student's *t*-test and ANOVA (Bonferroni post hoc test) were utilized for statistical analysis. Chi-square or Fisher's exact test was used for evaluating the correlations between MMP1 expression and various clinicopathological parameters. Survival data were used to establish the Kaplan–Meier curves, and the differences among the groups were analyzed by the log-rank test. Univariate and multivariate Cox regression analyses were performed to evaluate prognostic factors correlated with patients' overall survival. *P* values less than 0.05 were considered statistically significant.

## 3. Results

### 3.1. MMP1 mRNA Was Highly Expressed in HNSCC via Bioinformatics Analyses

**Figure 1:**
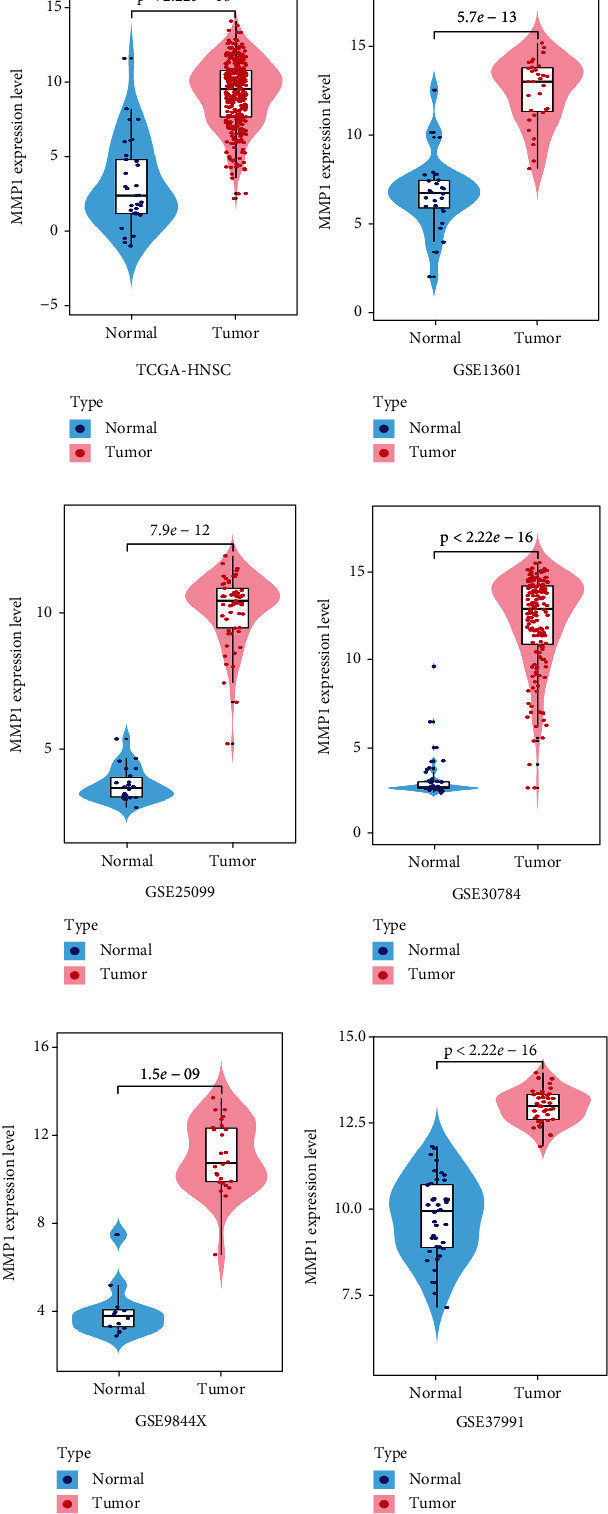
Overexpression of MMP1 mRNA in 6 HNSCC cohorts. (a–f) The mRNA levels of MMP1 (log2-transformed) were compared between HNSCC samples and normal counterparts in multiple patient cohorts. The original data were retrieved from GEO database and TCGA and then plotted using GraphPad Prism 8 software.

Accumulating evidence suggested that MMP1 was highly expressed in most cancers as well as pan-cancer analysis (Figure S1) from GEPIA2 [22, 23]. In order to investigate the expression level of MMP1 in tumor specimens of HNSCC patients, we preliminarily evaluated the mRNA expression level of MMP1 by interrogating the public gene expression database including TCGA and GEO. As shown in Figure 1(a), TCGA-HNSCC cohort showed that MMP1 mRNA expression was significantly upregulated in HNSCC samples compared to their non-tumor counterparts. Additionally, five independent HNSCC patients' cohorts from GEO database such as GSE13601, GSE25099, GSE30784, GSE9844X, and GSE37991 cohorts were utilized to identify MMP1 mRNA expression. As shown in Figures 1(b)–1(f), the abundance of MMP1 mRNA in HNSCC samples was also significantly increased in tumor samples. These figures indicated MMP1 might be involved in the occurrence and development of HNSCC.

### 3.2. MMP1 Overexpression Was Associated with Clinical Parameters in HNSCC Patients

**Figure 2:**
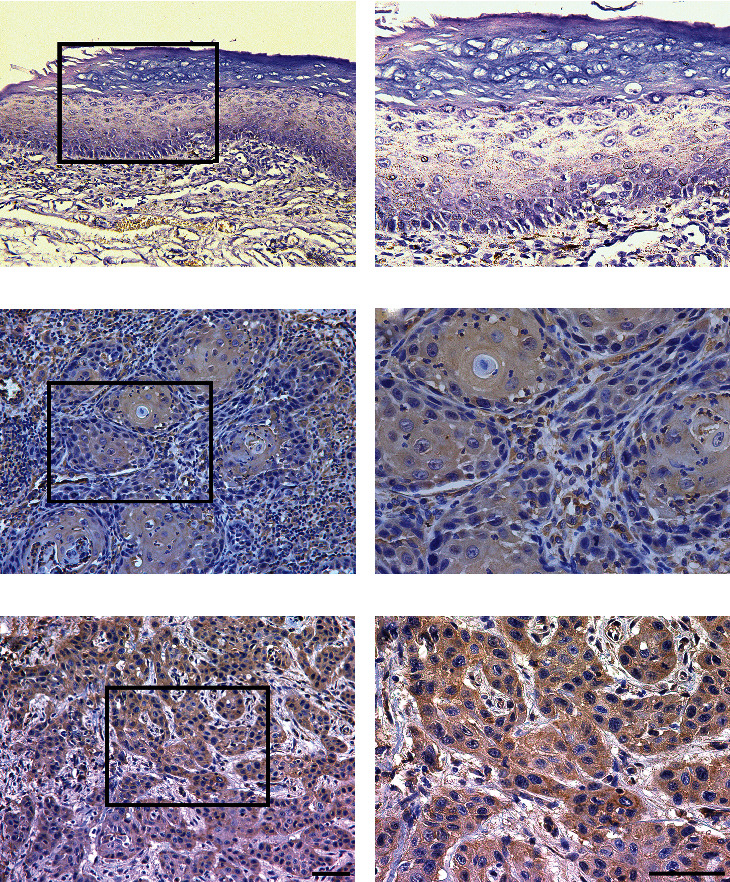
Immunohistochemical staining of MMP1 in human HNSCC samples. (a, b) Representative negative staining of MMP1 in normal oral epithelial. (c, d) Representative low expression of MMP1 in primary human HNSCC sample. (e, f) Representative high expression of MMP1 in primary human HNSCC sample. Nuclei are counterstained with hematoxylin. The areas marked by black box in the (a, c, e) images (upper panel) were shown in larger magnification as (b, d, f) images (lower panel), respectively. Scale bar: 100 *μ*m.

In order to further determine the expression pattern of MMP1 in HNSCC, we next performed immunohistochemical staining on 103 samples of primary HNSCC. The detailed demographic and clinicopathological parameters of these patients are listed in Table 1. In short, 54 males and 49 females were enrolled with an average age of 63.8 years. The follow-up time ranged from 4 to 95 months, with an average of 57.1 months. Until the last follow-up, 54 (52.4%) patients survived with disease-free, 9 (8.7%) patients still alive but with recurrences and/or cervical nodal metastases, and 40 (38.9%) patients died due to post-surgical recurrence, metastases, or other unrelated diseases. As shown in Figures 2(a)–2(f), rare MMP1 staining was observed in most healthy oral mucosa samples and a few HNSCC samples, whereas positive MMP1 cytoplasmic staining was detected in a variety of HNSCC samples. The expression patterns of MMP1 in HNSCC and normal oral epithelium were classified according to immunohistochemical scores. Consequently, MMP1 protein abundance could be categorized into low (40) or high expression (63) in HNSCC samples while negative (6), low (8), or high expression (6) in normal oral epithelial samples, which suggested that MMP1 was abnormally overexpressed in HNSCC (*P* < 0.0001, chi-square test). The relationships between MMP1 expression and clinical parameters of HNSCC patients are shown in Table 1. There were no significant associations between MMP1 expression and gender, age, smoking, alcohol drinking, and clinical stage. However, high MMP1 expression was positively associated with advanced tumor size, advanced pathological grade, and cervical node metastasis and with *P*-value 0.0097, 0.0006, and 0.0280, respectively.

### 3.3. Aberrant Overexpression of MMP1 Significantly Associated with Poor Prognosis in HNSCC Patients

**Figure 3:**
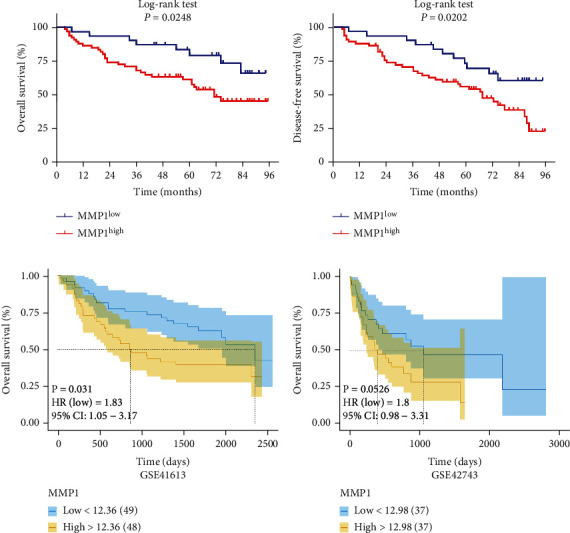
High MMP1 expression positively associates with reduced overall survival and disease-free survival rates in HNSCC patients. (a, b) Overall survival (a) and disease-free survival (b) analyses of patients stratified with high or low expression of MMP1 were estimated by the Kaplan-Meier method and compared with log-rank test. (c, d) Overall survival analyses of patients from GSE41613 (c) and GSE42743 (d) with high or low expression of MMP1 mRNA (median value as cutoff) were estimated by the Kaplan-Meier method and compared with log-rank test.

Next, the Kaplan-Meier survival analysis was utilized to investigate the correlation between MMP1 expression and patient prognosis. As shown in Figures 3(a) and 3(b), the outcome of the Kaplan-Meier analysis revealed that high expression of MMP1 was related to poor prognosis in overall survival and disease-free survival (log-rank test, *P* =0.0248, 0.0202). Moreover, the similar conclusion from GSE41613, GSE42743, and TCGA-HNSCC cohort also showed that overexpression of MMP1 had reduced overall survival compared to low MMP1 expression (Log-rank, *P* =0.031, 0.0526, 0,046, Figures 3(c) and 3(d) and Figure S2A). However, there were no correlations between MMP1 mRNA expression and disease-free survival in TCGA-HNSCC cohort, when patients were divided into low and high MMP1 expression subgroups using the median of MMP1 mRNA as cutoff (Figure S2B). We further investigated the associations between the abundance of MMP1 mRNA and pathological grade and clinical stage in TCGA-HNSCC. There were also no correlations between MMP1 mRNA expression and clinicopathological parameters (Figure S2C, D). Additionally, we evaluated the prognostic value of MMP1 expression in HNSCC by univariate and multivariate survival analysis. Consistent with the Kaplan-Meier survival analysis, univariate regression analysis also showed that MMP1 expression was significantly correlated with patient survival (*P* =0.023, Table 2). Moreover, multivariate regression analysis demonstrated MMP1 expression was identified as an independent factor of patient survival after adjusting for other demographic and clinicopathological parameters (*P* =0.014, Table 2).

### 3.4. Increased MMP1 Expression in Chemical-Induced HNSCC Tumorigenesis

**Figure 4:**
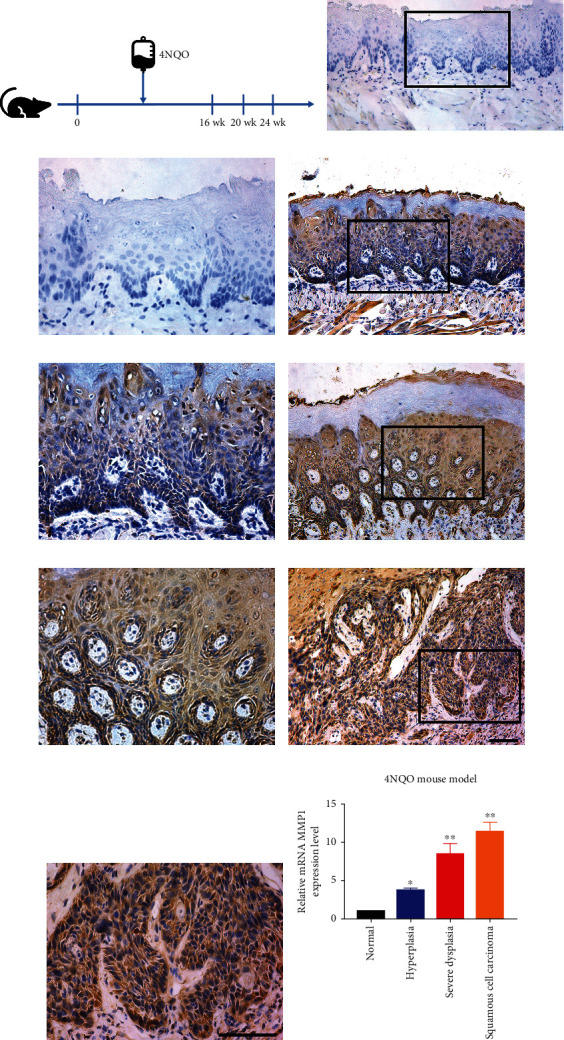
MMP1 expression pattern during HNSCC tumorigenesis in 4NQO-induced animal model. (a) Experimental scheme of 4NQO-induced HNSCC animal model. (b–i) Immunohistochemical staining of MMP1 in samples from diverse stages in 4NQO-induced animal model. Images in the upper panel (b, d, f, h) were representative staining of MMP1 in normal, epithelial with hyperplasia, epithelial with severe dysplasia/carcinoma in situ, and squamous cell carcinoma, respectively. Images in the lower panel (c, e, g, i) were magnified from the black box area in the (b, d, f, h) images in the upper panel, respectively. Scale bar: 100 *μ*m. (j) The mRNA levels of MMP1 during the 4NQO-induced HNSCC were measured by qRT-PCR in mice samples (*n* =5 samples per group). ^∗^*P* < 0.05, ^∗∗^*P* < 0.01, ANOVA analyses.

Based on our findings of high expression of MMP1 in human HNSCC samples, as shown in Figure 4(a), a well-established chemical-induced mouse model was utilized to further explore the expression of MMP1 in the occurrence and development of HNSCC. 4NQO treatment led to multiple lesions of the tongue including epithelial hyperplasia, dysplasia, carcinoma in situ, and squamous cell carcinoma, which largely reproduced the multi-stage tumorigenic biological behavior of HNSCC. As shown in Figures 4(b)–4(i), immunohistochemical staining of MMP1 in mouse specimens indicated significant cytoplasmic staining in dysplasia, carcinoma in situ, and invasive carcinoma, while negative or low staining in normal tongue epithelial. Positive MMP1 cytoplasmic staining was commonly observed in carcinoma (100%, 10/10), hyperplasia (60%, 6/10), and dysplasia/carcinoma in situ (60%, 6/10), but much less in samples with healthy mucosa (20%, 2/10). Moreover, consistent with IHC findings, qRT-PCR results revealed that the mRNA levels of MMP1 in carcinoma in situ/SCC samples were highly upregulated compared with normal tongue samples (Figure 4(j)). Above all, our findings demonstrated that MMP1 might be a putative oncogene driving the occurrence and development of HNSCC.

### 3.5. MMP1 Depletion Inhibited Cell Proliferation and Migration/Invasion and Promoted Apoptosis in HNSCC

**Figure 5:**
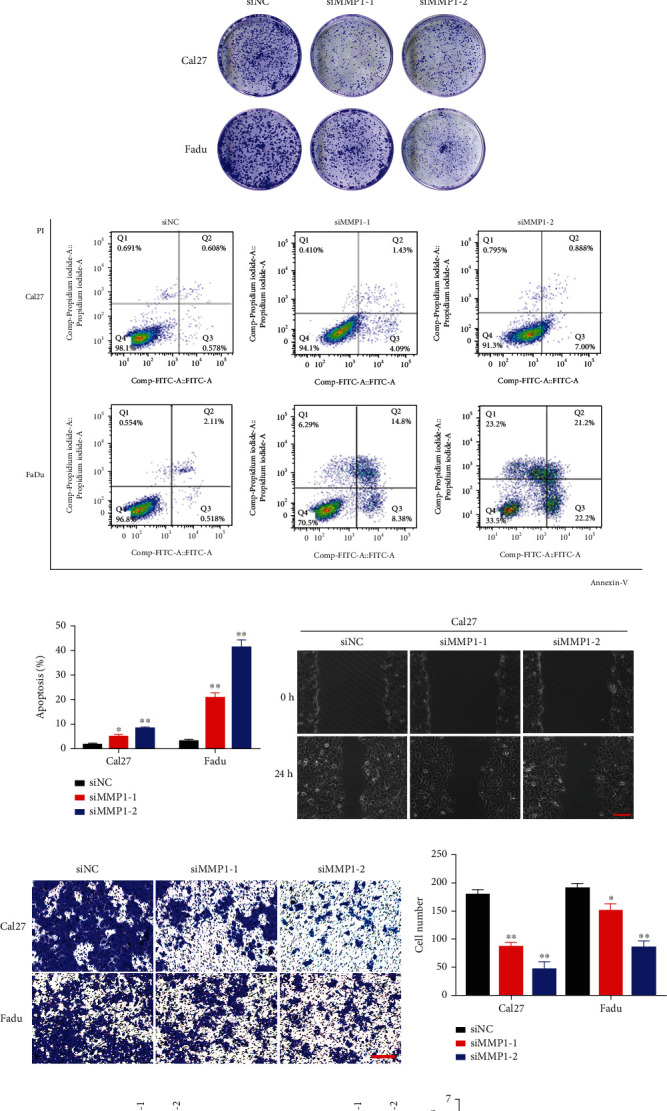
MMP1 knockdown inhibits cell proliferation and migration and invasion and triggers apoptosis in HNSCC cells. (a) Endogenous MMP1 protein expression was measured in a panel of HNSCC cell lines as compared to normal oral epithelial (HOK). Representative images of western blot (WB) were shown from 3 independent experiments. (b) Endogenous MMP1 was efficiently silenced by 2 siRNAs (siMMP1-1, siMMP1-2) in Cal27 and Fadu cells. Non-targeting siRNA was utilized as negative control (siNC). Representative images of WB are shown from 3 independent experiments. (c, d) Cell proliferation was remarkably suppressed when endogenous MMP1 was silenced as measured by CCK-8 viability assay. (e) The potentials of colony formation were significantly inhibited in MMP1-depleted cells as compared to control (siNC). (f, g) Increased percentages of cell undergoing apoptosis were evident following MMP1 knockdown as assayed by Annexin V-PI staining. (h, i) The migration (G) and invasion (H) abilities were significantly reduced in siMMP1-transfected cells in wound healing and transwell assays, respectively. (k–m) The protein and mRNA abundance of migration/invasion relevant marker E-cadherin, N-cadherin, Vimentin, and Snail was compared in cells infected siMMP1 or control siNC. (n) Correlation between generic EMT score for HNSCC samples from TCGA dataset and MMP1 expression. Generic EMT score was calculated following the method of single-sample Gene Set Enrichment Analysis (ssGSEA) [24]. Scale bar: 100 *μ*m, Data shown here are mean ± SD from three independent experiments, ^∗^*P* < 0.05, ^∗∗^*P* < 0.01, ANOVA analyses.

The above experiments have demonstrated the tumor-promoting effect of MMP1; we next aimed to elucidate its oncogenic roles by siRNA-mediated loss of function approach during HNSCC initiation and progression. We first detected the expression of MMP1 in a group of HNSCC cell lines and found that MMP1 protein abundance was highly overexpressed in all HNSCC cell lines compared with HOK cells (Figure 5(a)). We next selected Cal27 and Fadu cells for siRNA-mediated knockdown experiments owing to relatively higher MMP1 protein in them. By introducing two independent siRNAs targeting human MMP1 into Cal27 and Fadu cells, the subsequent changes of MMP1 expression and cell phenotype were monitored. As shown in Figure 5(b), after siRNA transfection, MMP1 protein expression was significantly decreased in Cal27 and Fadu cells. Then, we utilized these transfected cells for phenotypic experiments. CCK-8 viability assay and colony formation assay indicated significantly lower proliferation rate in Cal27 and Fadu cells following MMP1-siRNA transfection (Figures 5(c)–5(e)). In addition, cytometric assay showed that the proportions of apoptotic cells in siMMP1-transfected cells were significantly increased from 1.3% to 5.0/8.3% in Cal27 and from 2.6% to 20.8/41.3% in Fadu, respectively (Figures 5(f) and 5(g)). Both wound healing and transwell invasion assays demonstrated the migratory and invasive abilities of cells following MMP1 knockdown were significantly reduced (Figures 5(f)–5(i)). In line with these findings, western blot and qRT-PCR results indicated the protein and mRNA expression of EMT/metastasis-associated marker Vimentin, N-cadherin, and Snail were downregulated concomitant with E-cadherin upregulated following MMP1 knockdown (Figures 5(k)–5(m)). Finally, we generated generic EMT signature to score the EMT status of these samples from TCGA-HNSCC cohort, which was a useful tool for objective, systematic investigation of EMT roles and dynamics in cancer progression, treatment response, and survival [24]. Consistent with the above findings, our results from this scoring system revealed that MMP1 expression was significantly associated with EMT score in TCGA-HNSCC (*R* =0.322, *P* < 0.0001, Figure 5(n)). These results suggested that MMP1 knockdown inhibited cell proliferation and migration/invasion and activates apoptosis in HNSCC by EMT.

### 3.6. Functional Enrichment Analysis of the Differentially Expressed Genes (DEGs) Correlated to MMP1

**Figure 6:**
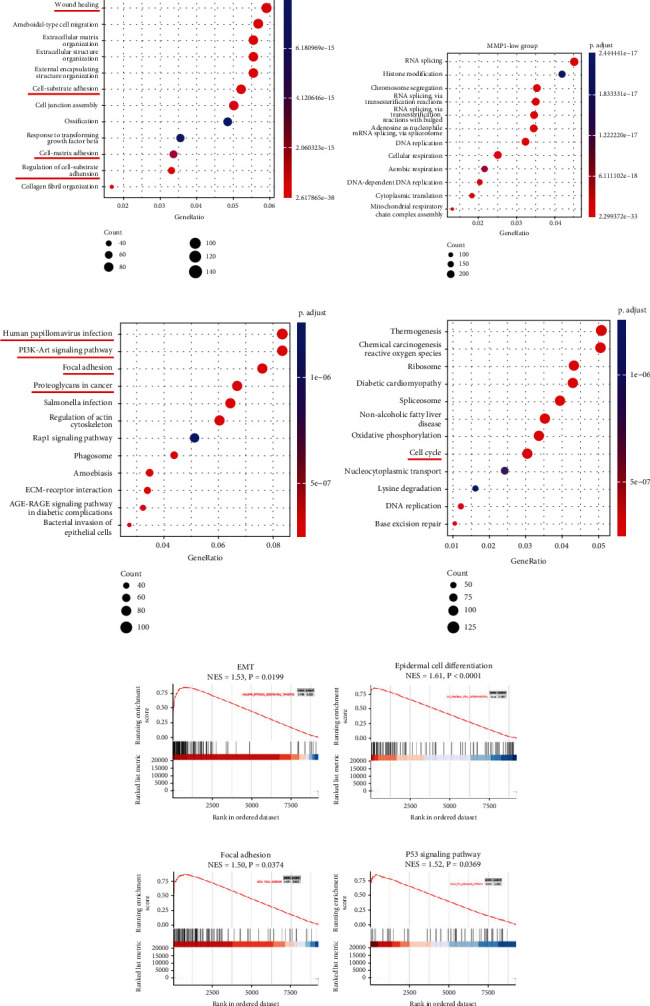
The GO, KEGG, and GSEA analyses results for the differentially expressed genes between high-MMP1 and low-MMP1 groups. (a, c) GO (upper panel) and KEGG (low panel) analysis for the correlated genes in MMP1-high group. (b, d) GO (upper panel) and KEGG (low panel) analysis for the correlated genes in MMP1-low group. (e–h) Functional annotations of DEGs between two groups by GSEA analyses revealed high enrichment in several cancer-relevant categories including enriched in EMT pathway (e), epidermal cell differentiation (f), focal adhesion (g), and P53 signaling pathway (h). GSEA enrichment plots with normalized enrichment score (NES) and *P*-values were shown.

Next, we utilized functional enrichment analysis to complement the in vitro loss-of-function experiment in exploring MMP1 pro-tumorigenic functions. Firstly, we screened 26526 negative and 10403 positive genes correlated to MMP1 from TCGA-HNSCC on GEPIA 2 (Figure S3A). Then, we performed GO and KEGG analyses on these genes. The data suggests that MMP1 correlated genes enriched in numerous cancer-related biofunction and pathways such as wound healing, cell-substrate adhesion, cell-matrix adhesion, PI3K-Akt signaling pathway, human papillomavirus infection, focal adhesion, MAPK signaling pathway, and proteoglycans in cancer, indicating MMP1 played a pro-tumorigenic role by these pathways (Figure S3B, C). We further divided the expression of MMP1 into high-expression group and low-expression group (median as cutoff) by the method of bipartition in TCGA-HNSCC data. Then, we utilized bioinformatics analysis to recognize the nominee genes that might be correlated with high/low group of MMP1 (Figure S3D–S3F). Consistent with the above findings, GO and KEGG analysis for the correlated genes associated with high group of MMP1 were significantly enriched in wound healing, cell-substrate adhesion, cell-matrix adhesion, PI3K-Akt signaling pathway, human papillomavirus infection, focal adhesion, and proteoglycans in cancer (Figures 6(a) and 6(c)). Additionally, KEGG and GO analyses showed the correlated genes associated with low group of MMP1 were related to cell cycle and metabolism (Figures 6(b) and 6(d)). Finally, we analyzed the differentially expressed genes (DEGs) between two groups using GSEA analysis. The results revealed that DEGs between two groups were significantly enriched in EMT pathway (Figure 6(e)), epidermal cell differentiation (Figure 6(f)), focal adhesion (Figure 6(g)), and p53 signaling pathway (Figure 6(h)). Above all, combined with cell assay in vitro and bioinformatics results extremely support the idea that MMP1 is an oncogene promoting cell migration and invasion by EMT in HNSCC.

## 4. Discussion

MMPs have been long appreciated as important mediators of tissue remodeling, targeting for proteolysis numerous extracellular matrix components. Previous studies have reported MMPs play essential roles including tumor growth, differentiation, apoptosis, angiogenesis, migration, invasion, and immune surveillance in multiple steps of cancer progression [25, 26]. MMP1 as a critical member of the MMP family, a variety of evidence have reported its oncogenic roles and prognostic significance underlying multiple cancer contexts [9, 15, 27, 28]. Herein, we used public databases, HNSCC samples, vitro loss-of-function experiments, and bioinformatics analysis to determine the expression form, prognostic significance, and carcinogenic effect of MMP1 in HNSCC. The results demonstrate that MMP1 as a putative oncogene promotes HNSCC initiation and development and also be a novel biomarker with clinical translation potential.

Accumulating evidence has revealed that MMP1 is usually overexpressed in multiple cancers including lung cancer, esophageal squamous cell carcinoma (ESCC), nasopharyngeal carcinoma, breast cancer, and OSCC [9, 16, 27, 29, 30]. Consistent with previous studies, our results revealed that MMP1 was highly expressed in multiple independent patient cohorts from publicly available dataset and immunohistochemistry in primary HNSCC samples. In line with this findings, the results of 4NQO-induced HNSCC model showed that the expression of MMP1 increased with the occurrence and progression from hyperplasia to invasive carcinoma. Although MMP1 amplification was identified in multiple cancers, the mechanisms of its overexpression were still unclear. Therefore, we utilized cBioPortal to explore genetic alteration of MMP1 and found genetic amplication occurred in only 5% HNSCC sample (Figure S4), thus largely ruled out the possibility of MMP1 genetic amplification responsible for its overexpression in most HNSCC samples. The accurate mechanisms of its amplication in HNSCC still need further investigation. All these findings strongly demonstrate MMP1 promotes HNSCC initiation and development as a bona fide oncogene.

Several previous reports have proposed important clinical relevance of MMP1 overexpression in human cancer. For example, elevated MMP1 was significantly associated with pN status and clinical stage in ESCC [16]. In colorectal cancer, MMP1 overexpression was positively correlated with TNM stage and lymphatic metastasis [11, 31]. Moreover, abnormal overexpression of MMP1 was positively correlated with the worse outcome in breast cancer, ESCC, melanoma, colon cancer, and pancreatic carcinoma [12–14, 16, 29]. In line with these findings, our results from primary HNSCC samples revealed that MMP1 overexpression was significantly associated with advanced tumor size, cervical node metastasis, and advanced pathological grade. In addition, both the Kaplan-Meier survival and univariate/multivariate Cox regression analyses showed that the overexpression of MMP1 positively correlated with reduced survival, and it could be served as an independent prognostic predictor for patients' survival. Complementary, TCGA-HNSCC and GEO data also demonstrated that overexpression of MMP1 was associated with decreased overall survival which further strengthened our results. However, the mRNA expression of MMP1 was not related to clinical grades, pathological stages, and disease-free survival in TCGA-HNSCC cohort. We reasoned that it is conceivable because of the significant heterogeneity of HNSCC between TCGA cohort and our cohort. Secondly, different classification methods of TCGA-HNSCC cohort and our cohort and the inconsistency between mRNA and protein expression of MMP1 might lead to this discrepancy. Of course, more centers and more patients are needed to establish the clinical and prognostic significance of MMP1 in HNSCC, so as to determine whether MMP1 can be used as novel biomarker for evaluation of patients' prognosis.

It is undisputed that MMP1 is critically involved in tumorigenesis by promoting cell proliferation, migration, invasion, EMT, and inhibiting apoptosis [11, 15, 32]. For example, the downregulation of MMP1 inhibited invasion and migration in gastric cancer and cervical cancer [33, 34]. In addition, in colorectal cancer and ESCC, the overexpression of MMP1 promoted cell proliferation, migration, invasion, EMT in vitro, and metastasis in vivo [11, 16]. The latest research demonstrated knockdown of MMP1 inhibited cell growth, migration, and phosphorylation of AKT in Fadu cell lines. Moreover, absence of MMP1 decreased tumor migration but not growth in vivo [17]. Consistent with these studies, our results demonstrate that MMP1 has a variety of tumorigenic roles by promoting cell proliferation, migration, and invasion and suppressing apoptosis in HNSCC cells. There are also a few works in HNSCC involving MMP1. These studies mainly indicated that MMP1 was the downstream target gene involving HNSCC invasion and metastasis [35–37]. However, these studies have not explained in detail the carcinogenic function of MMP1 itself compared with our results.

Accumulating evidence has shown EMT is a significant approach regulating migration and invasion necessary for primary tumor cells in recent years [18, 38]. Noticeably, our results demonstrated that MMP1 knockdown strongly inhibited invasion and motility by facilitating MET in HNSCC as evidenced by the mRNA and protein expression of EMT-related markers were reversed upon MMP1 depletion as well as EMT scoring system. Moreover, bioinformatics analysis from public databases data sources also suggested MMP1 associated differentially expressed genes were significantly rich in EMT pathway and wound healing. These results partly explained the correlation between the high expression of MMP1 and cervical lymph node metastasis. In addition, bioinformatics analysis results also indicated MMP1 has involved in significant cancer-related pathways such as PI3K-Akt signaling pathway, focal adhesion, MAPK signaling pathway, and cell cycle. For instance, knockdown of MMP1 inhibited the progression of colorectal cancer by suppressing the PI3K-Akt-myc signaling pathway and EMT [11, 39]. Additionally, Ji et al. have demonstrated the downregulation of MMP-1, MMP-2, and MMP-7 could play anti-metastatic effects of via inactivation of MAPK signaling and induction of focal adhesion formation in hepatocellular carcinoma [40]. Complementary, Yu et al. have reported that MMP1 significantly facilitated colon cancer cell proliferation via accelerating cell cycle transition from G0/G1 to S and G2/M phase [39], which in part strengthened our data. Collectively, our findings together with others strongly suggest that MMP1 probably functions as a pro-tumorigenic oncogene via enhancing cancer cell proliferation, migration, and invasion in HNSCC by inducing EMT and cancer-related pathways. However, the precise molecular mechanisms regulating EMT await further elucidation.

## 5. Conclusion

Our findings revealed the expression pattern and prognostic and tumorigenic roles of MMP1 and identified MMP1 as a novel biomarker with diagnostic and prognostic significance in HNSCC. However, the limited number of patients examined and the retrospective nature of our study make it impossible to clearly prove the prognostic and diagnostic significance of MMP1 in HNSCC. So that more centers and more patients are needed to establish its prognostic and diagnostic utility in HNSCC. In addition, our findings also demonstrated MMP1 as a putative oncogenic mediator underlying HNSCC initiation and progression, which suggested that selective targeting of MMP1 by genetic or chemical methods may have translation prospects, but in order to further explore this, more work needs to be done in the future to find accurate MMP1 regulatory networks and novel inhibitors against MMP1.

## Figures and Tables

**Table 1:** The associations between MMP1 expression and multiple clinicopathological parameters in HNSCC samples.

Clinicopathological parameters	Cases	MMP1		*P*-values
		Low	High	
Gender	103	40	63	
Male	54	23	31	0.4269
Female	49	17	32	
Age				
≤60	43	18	25	0.6829
>60	60	22	38	
Smoking				
No	75	30	45	0.8211
Yes	28	10	18	
Alcohol use				
No	61	20	41	0.1526
Yes	42	20	22	
Tumor size				
T1-T2	69	33	36	**0.0097** ^∗^
T3-T4	34	7	27	
Pathological grade				
I	66	34	32	**0.0006** ^∗^
II	17	5	12	
III	20	1	19	
Cervical node metastasis				
N(0)	71	33	38	**0.0280** ^∗^
N(+)	32	7	25	
Clinical stage				
I	18	8	10	0.2842
II	53	20	33	
III	20	10	10	
IV	12	2	10	

^∗^ indicate statistical significance with *P*-values less than 0.05.

**Table 2:** Univariate and multivariate survival analyses (proportional hazards method) for patients with primary HNSCC.

Variable	Univariate survival analysis			Multivariate survival analysis		
	Hazard ratio	95% CI	*P*-value	Hazard ratio	95% CI	*P*-value
Gender (male, female)	1.132	0.558-2.299	0.731	1.070	0.334-3.423	0.909
Smoking (no, yes)	1.095	0.504-2.378	0.819	1.389	0.469-4.108	0.553
Alcohol use (no, yes)	0.802	0.389-1.654	0.550	0.648	0.205-2.047	0.460
Age (≤60, >60)	1.192	0.561-2.535	0.648	1.588	0.730-3.452	0.243
Tumor size (T1-T2, T3-T4)	0.675	0.236-1.931	0.463	0.575	0.199-1.660	0.306
Pathological grade (I, II-III)	1.307	0.871-1.961	0.196	1.206	0.779-1.867	0.838
Cervical nodal metastasis (N0, N+)	1.402	0.628-3.133	0.410	1.816	0.483-6.832	0.377
Clinical stage (I-II, III-IV)	1.302	0.548-4.133	0.549	1.273	0.788-2.198	0.641
MMP1 expression (low, high)	3.306	1.163-7.928	**0.023** ^∗^	3.410	1.281-9.082	**0.014** ^∗^

^∗^ indicate statistical significance with *P*-values less than 0.05.

## Data Availability

Data to support the findings of this study is available on reasonable request from the corresponding author.

## Supplementary Materials

Supplementary Materials Authors are responsible for providing the final supplementary materials files that will be published along with the article. Figure S1-S4 legends to supplement and demonstrate the corresponding conclusions in my article.
