# Supplementary material for: MMP1 Overexpression Promotes Cancer Progression and Associates with Poor Outcome in Head and Neck Carcinoma
Source: Comput Math Methods Med. 2022 Sep 5;2022:3058342. doi: 10.1155/2022/3058342 (PMC9467809; doi:10.1155/2022/3058342)
Supplement: Supplementary Materials — Authors are responsible for providing the final supplementary materials files that will be published along with the article. Figure S1-S4 legends to supplement and demonstrate the corresponding conclusions in my article. [file 3058342.f1.docx]

**Figure S1**


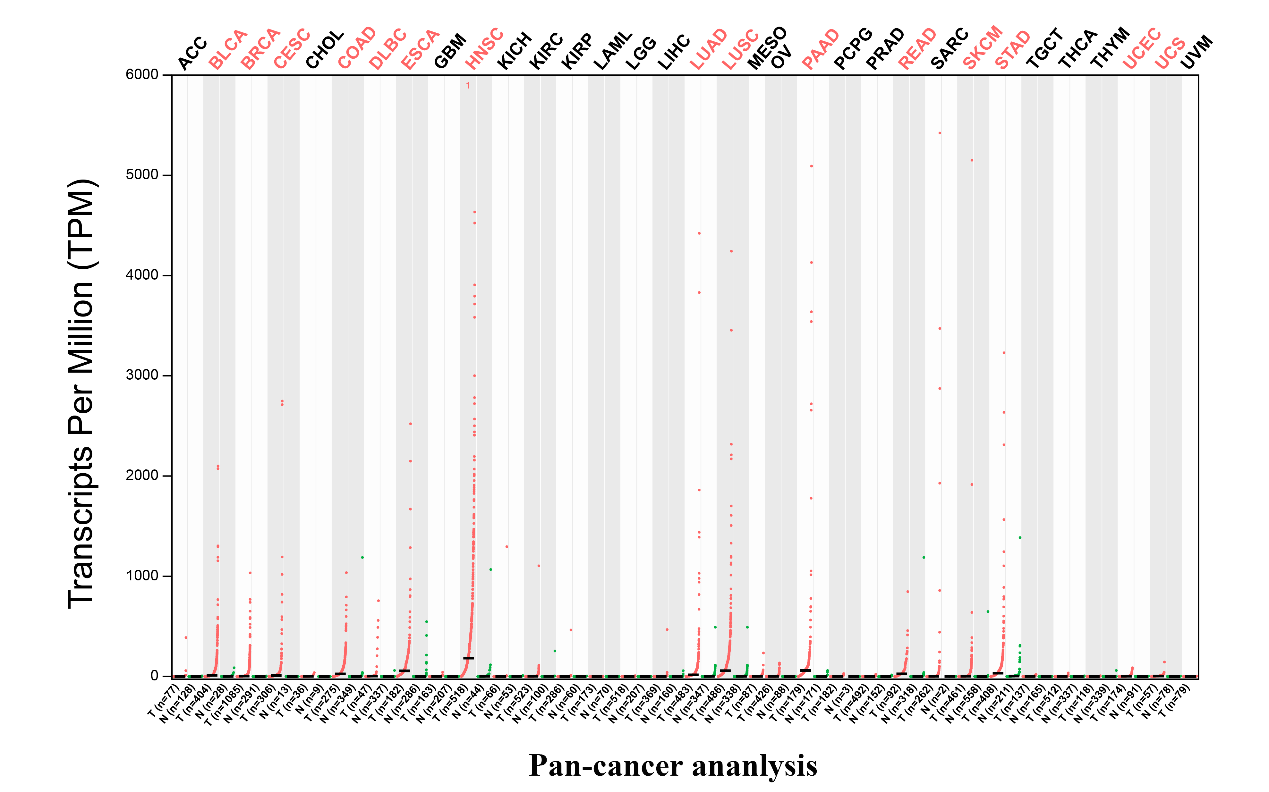


**Figure S1.** MMP1 mRNA expression pattern in pan-cancer analysis from GEPIA2.

**Figure S2**


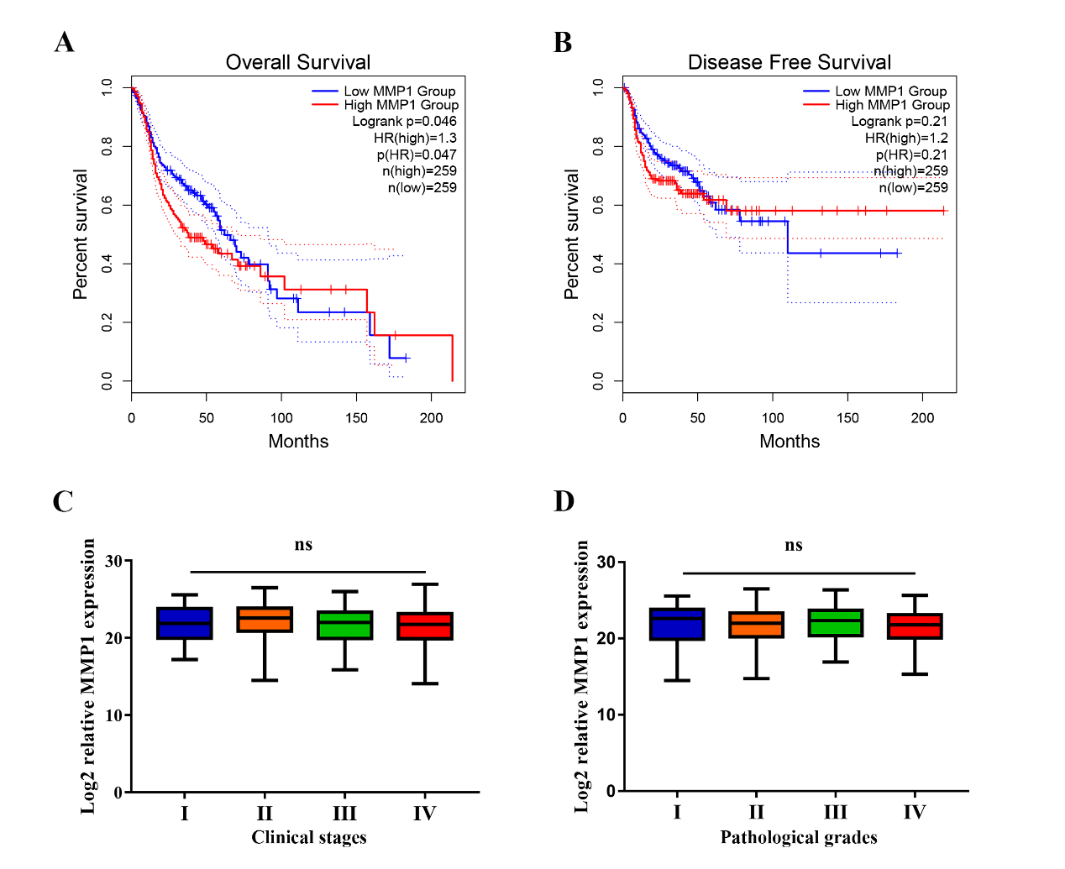


**Figure S2.** The associations between MMP1 expression and clinical parameters in TCGA-HNSCC cohort.

**A, B:** Overall and disease-free survival analyses of TCGA-HNSCC patients with high or low expression of TEAD4 mRNA (median value as cutoff) were estimated by Kaplan-Meier method and compared with Log-rank test; **C:** Relative expression of MMP1 mRNA (Log2-transformed) was compared TCGA-HNSCC subgroups stratified by clinical stage. NS denotes not significant difference between groups. Y-axis represents the median intensity, 25th, and 75th percentile data; **D:** Relative expression of MMP1 mRNA (Log2-transformed) was compared in TCGA-HNSCC subgroups stratified by pathological grades. Y-axis represents the median intensity, 25th, and 75th percentile data.

**Figure S3**


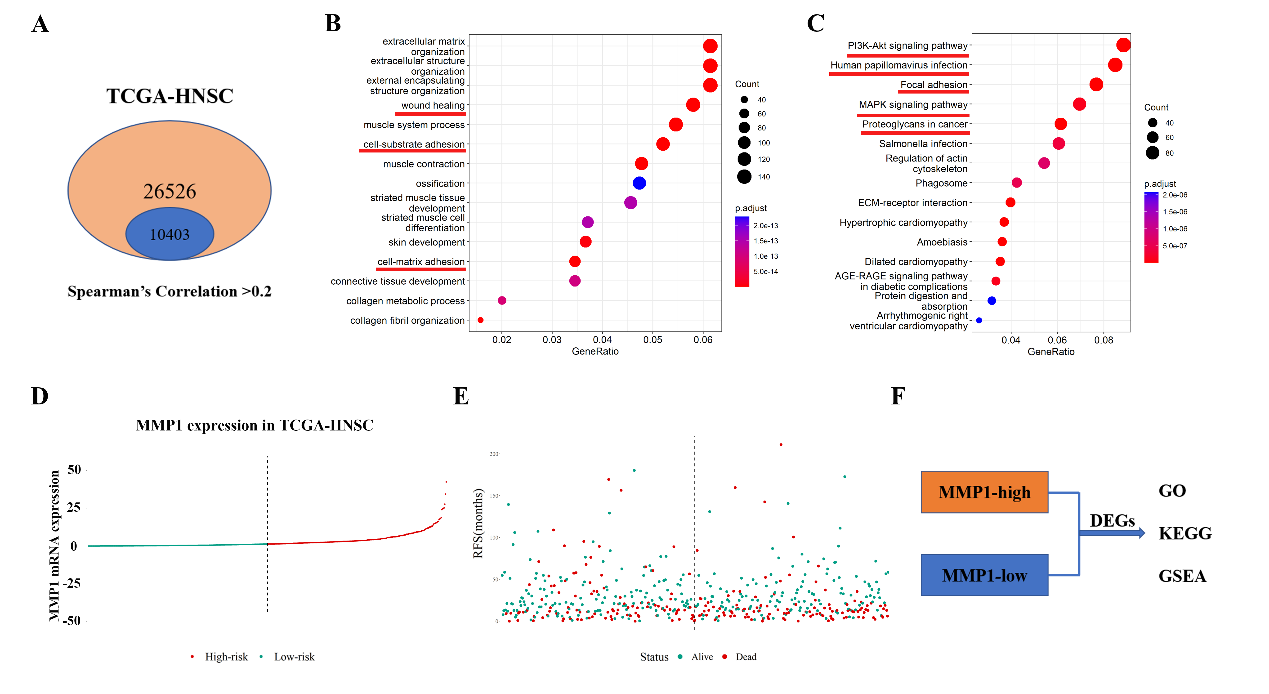


**Figure S3.** The biofunction of MMP1 correlation genes.

**A:** Schematic diagram of the numbers of MMP1-associated-genes with Spearman’s correlation > 0.2 subset; **B, C:** The top 15 involved significant biological process (**GO, B**) and pathways (**KEGG, C**) in these positive correlated genes; **D-E:** Scatter plot (**D**) displaying the MMP1 level of each patient from TCGA-HNSCC dataset. According to the median-cutoff MMP1 expression, patients were divided into high or low expression groups and survival status plot (**E**) shows the patients with the high or low group; **F:** Schematic diagram of further bioinformatic analysis between high or low group of patients.

**Figure S4**


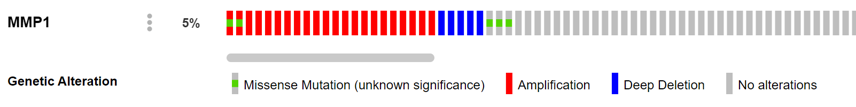


**Figure S4.** MMP1 genetic alternation in HNSCC-cohort from cBioPortal platform.
